# Supplementary material for: Differential Response of Wheat Rhizosphere Bacterial Community to Plant Variety and Fertilization
Source: Int J Mol Sci. 2022 Mar 25;23(7):3616. doi: 10.3390/ijms23073616 (PMC8998456; doi:10.3390/ijms23073616)
Supplement: Supplementary file 1 [file ijms-23-03616-s001.zip › Supplemental information file.pdf]

Differential Response of Wheat Rhizosphere Bacterial Community to Plant Variety and Fertilization

Lisa Cangioli [1], Marco Mancini [2], Marco Napoli [2,*], Camilla Fagorzi [1], Simone Orlandini [2], Francesca Vaccaro [1], and Alessio Mengoni [1,*]

Supplementary information file

Dataset S1: Table reporting the amplicon sequence variants (ASVs) detected, their taxonomic assignment and occurrence; .csv file.

Table S1: Table containing, for each sample, the number of total input reads from fastq files and the number of reads that passed the filtering parameters; .csv file.

Table S2: Table of Good's coverage values. Good's coverage was estimated on the amplicon sequence variant (ASV) table for each sample after removing chimeras; .csv file.

Table S3: List of richness, diversity and evenness indices; .csv file.

Table S4: List of p values calculated through a Permutational Multivariate Analysis of Variance (PERMANOVA) test performed on the phyloseq object after a centered log ratio (CLR) transformation; .csv file.

Table S5: Results of DeSeq2 on ASVs mostly differing among varieties; .csv file.

Table S6: List of all identified metabolic pathways present in the taxa retrieved in the bacterial community. A total of 422 pathways, all distributed in all samples was detected; .csv file.

Table S7: Scott–Knott test based on the averages the relative gene quantification with respect to 16S rRNA gene (Ct target specific gene)/Ct 16S rRNA gene) for each gene target in relation to wheat variety and N and P treatment; . xls file

Table S8: List of primers used for the quantitative PCR on *amoA*, *nifH*, *nirK* and *nosZ* genes; . xls file

Figure S1: Rarefaction curve of all soil samples. The x axis indicates the number of sequences found for each sample, while the y axis indicates the species richness;

Figure S2: Bar plots showing the relative abundances of bacterial phyla grouped by N and P treatment in relation to (a) soil type and (b) wheat varieties;

Figure S3: Principal Coordinate Analysis (or Metric Multidimensional Scaling) to the centered log-ratio transformation;

Figure S4: Total number of reads from ASVs belonging to Blastocatellales and Pyrinomonadales.

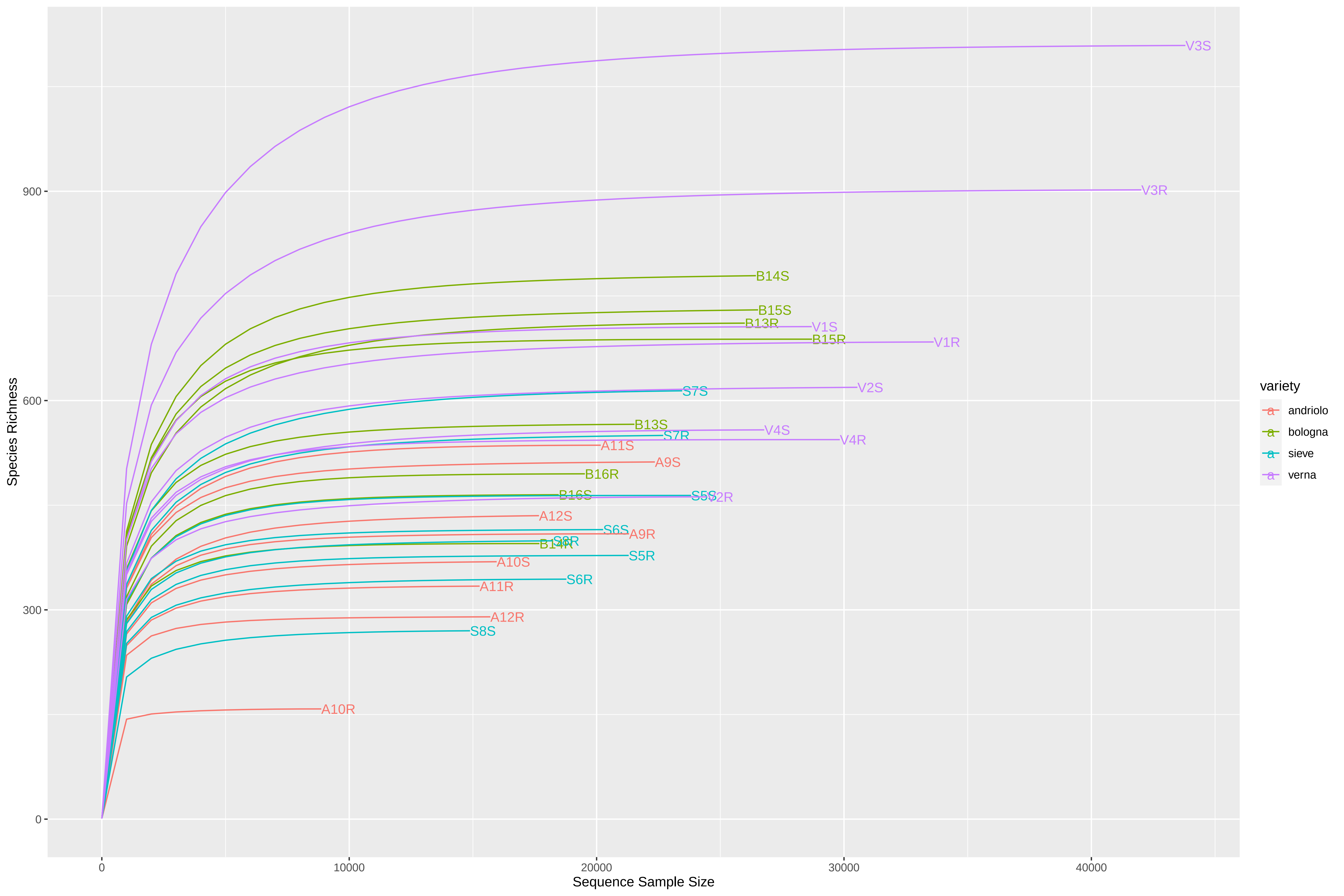

Species Richness
Sequence Sample Size
0
300
600
900
0
10000
20000
30000
40000
variety
andriolo
bologna
sieve
verna
V3S
V3R
B14S
B15S
B13R
V1S
B15R
V1R
V2S
S7S
B13S
V4S
S7R
V4R
A11S
A9S
B16R
B16S
S5S
V2R
A12S
S6S
A9R
S8R
B14R
S5R
A10S
S6R
A11R
A12R
S8S
A10R

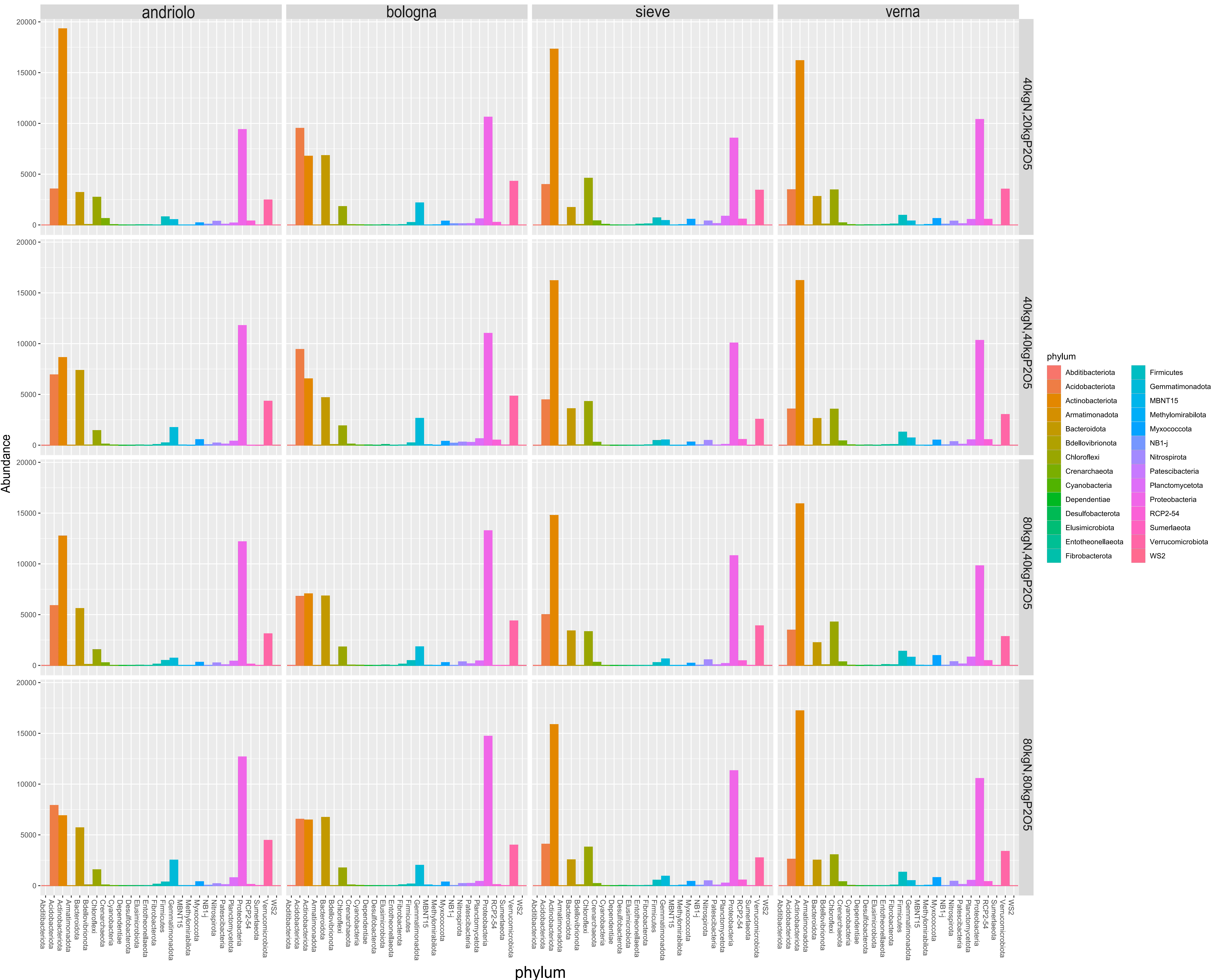

andriolo
bologna
sieve
verna
40kgN,20kgP2O5
40kgN,40kgP2O5
80kgN,40kgP2O5
80kgN,80kgP2O5
Abundance
phylum
0
5000
10000
15000
20000
Abditibacteriota
Acidobacteriota
Actinobacteriota
Armatimonadota
Bacteroidota
Bdellovibrionota
Chloroflexi
Crenarchaeota
Cyanobacteria
Dependentiae
Desulfobacterota
Elusimicrobiota
Entotheonellaeota
Fibrobacterota
Firmicutes
Gemmatimonadota
MBNT15
Methylomirabilota
Myxococcota
NB1-j
Nitrospirota
Patescibacteria
Planctomycetota
Proteobacteria
RCP2-54
Sumerlaeota
Verrucomicrobiota
WS2

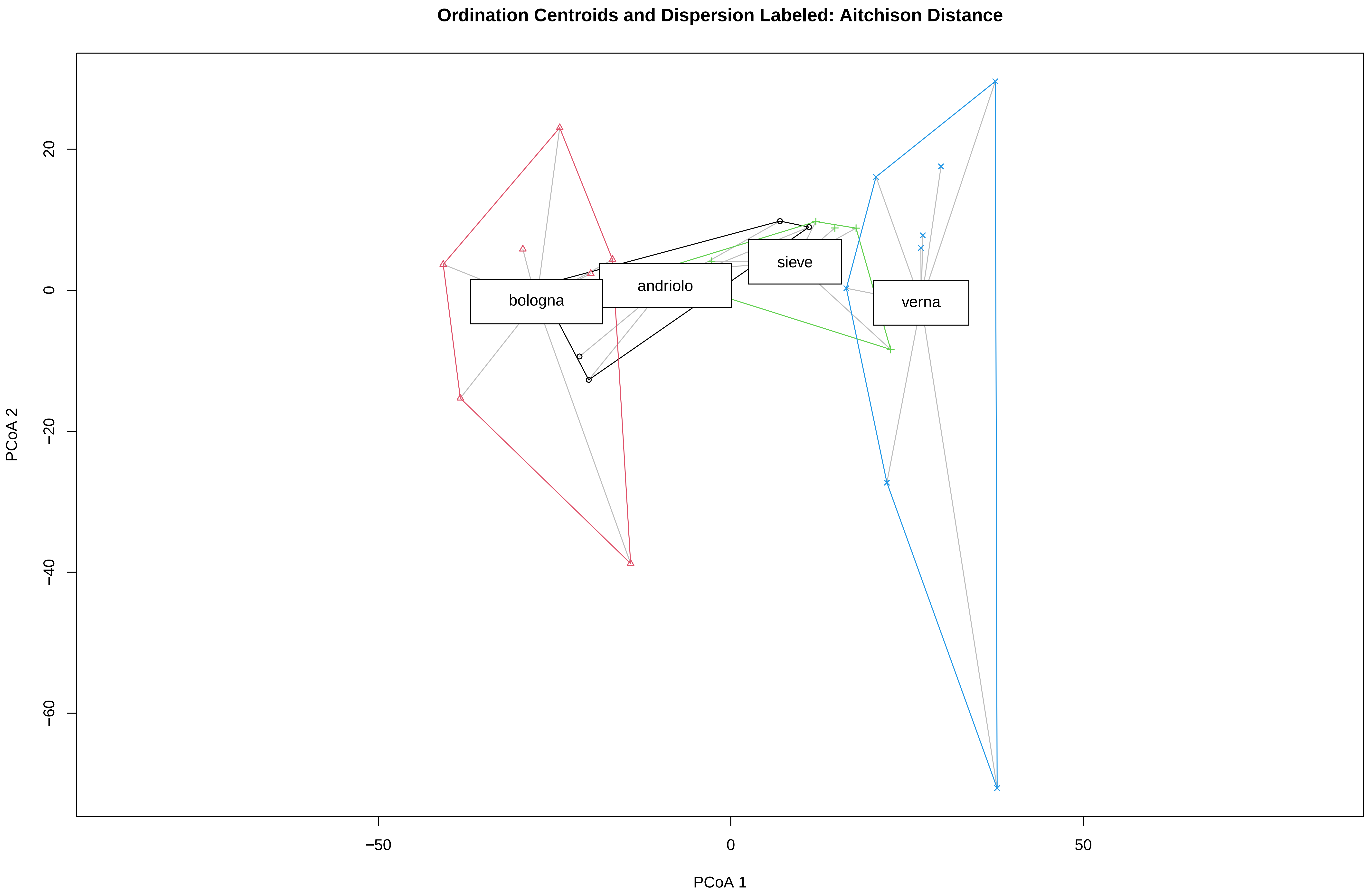

Ordination Centroids and Dispersion Labeled: Aitchison Distance
PCoA 1
PCoA 2
−50
0
50
20
0
−20
−40
−60
bologna
andriolo
sieve
verna

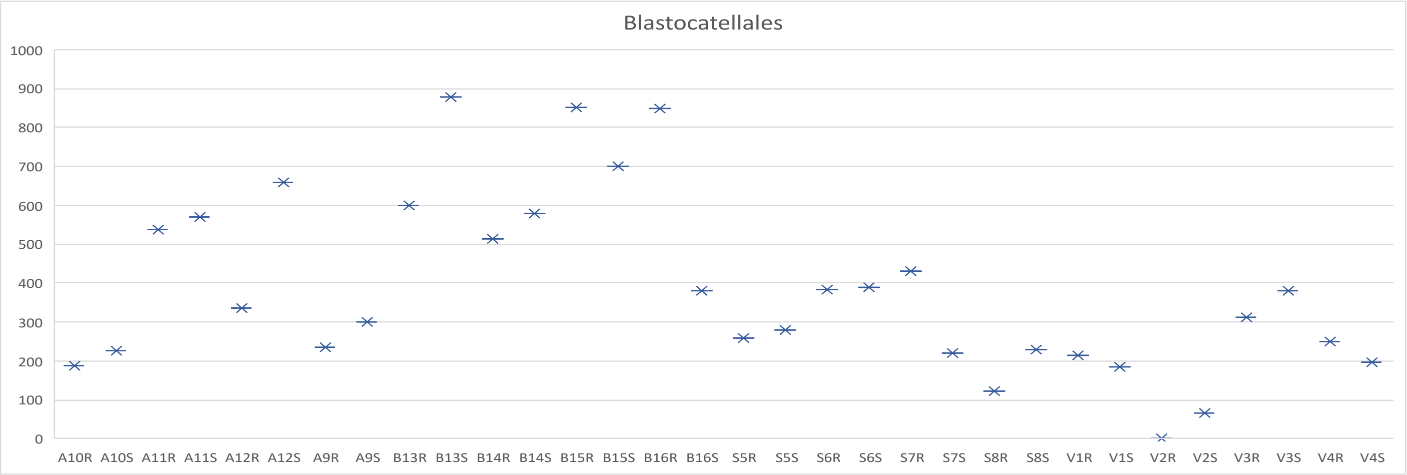
Blastocatellales
1000
900
800
700
600
500
400
300
200
100
0
A10R A10S A11R A11S A12R A12S A9R A9S B13R B13S B14R B14S B15R B15S B16R B16S S5R S5S S6R S6S S7R S7S S8R S8S V1R V1S V2R V2S V3R V3S V4R V4S

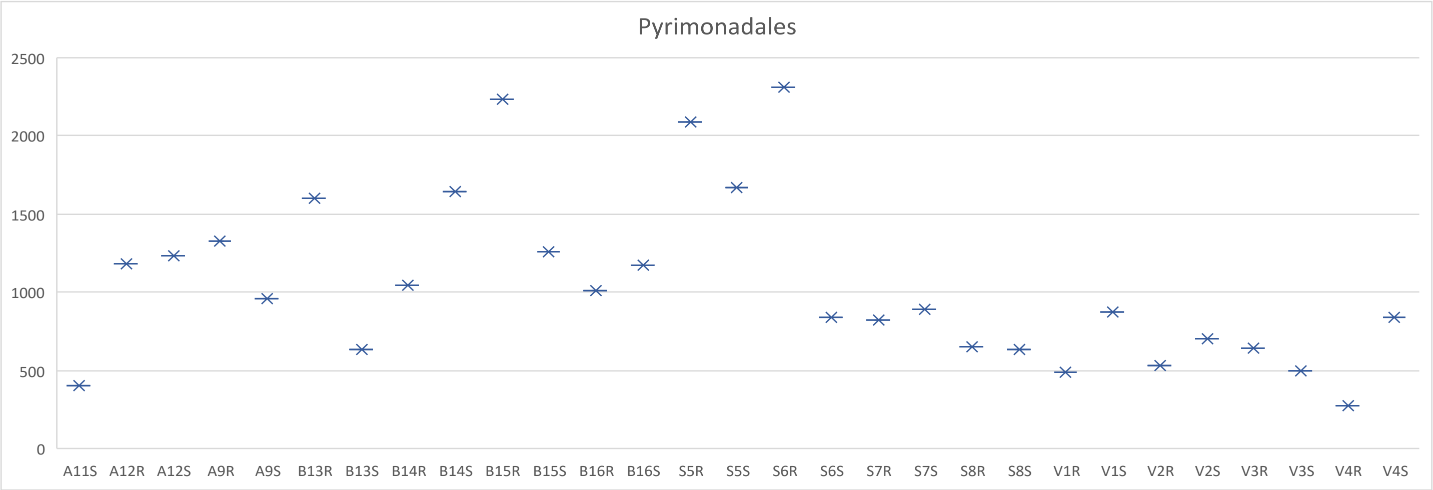
Pyrimonadales
2500
2000
1500
1000
500
0
A11S A12R A12S A9R A9S B13R B13S B14R B14S B15R B15S B16R B16S S5R S5S S6R S6S S7R S7S S8R S8S V1R V1S V2R V2S V3R V3S V4R V4S
